# Learning engagement as a protective moderator of the admission score–vocational course achievement association

**DOI:** 10.3389/fpsyg.2026.1971032

**Published:** 2026-10-07

**Authors:** Xiuzhen Fan, Maoguo Ye

**Affiliations:** 1 Sehan University, Yeongam-gun, Jeollanam-do, Republic of Korea; 2 Panyu Vocational Technical School, Guangzhou, Guangdong, China; 3 Heyuan Polytechnic, Heyuan, Guangdong, China

**Keywords:** academic self-efficacy, admission scores, learning engagement, protective moderation, teacher support, vocational course achievement, vocational higher education

## Abstract

**Introduction:**

Admission scores predict college performance, but engagement may alter this association. We tested learning engagement as a protective moderator and examined related indirect associations. Methods: We matched admission records, a May–June 2026 survey, and same-semester grades extracted July 13, 2026 for 491 Chinese vocational college students; the survey-to-extraction interval was 14–45 days.

**Results:**

Engagement weakened the admission-score–achievement association (*B* = −0.263, *p* < 0.001; standardized interaction = −0.144, 95% bootstrap CI [−0.214, −0.071]; ΔR^2^ = 0.021). The high-engagement slope was imprecise (*B* = 0.099, *p* = 0.078), not demonstrated to be zero; 18.1% of students exceeded the Johnson–Neyman threshold. Self-efficacy showed cross-sectional indirect associations with achievement (ab = 0.321, 95% bootstrap CI [0.232, 0.416]) and career adaptability (ab = 0.153, 95% bootstrap CI [0.106, 0.202]) through engagement. Teacher support modestly moderated the engagement–achievement association (*B* = 0.348, *p* = 0.025; ΔR^2^ = 0.009). Results remained stable in integrated and course-adjusted analyses.

**Discussion:**

Engagement was associated with weaker dependence of achievement on admission scores, but the interactions were small and observational. The indirect pathways and teacher-support interaction require independent replication.

## 1 Introduction

Admission scores are among the most commonly used sources of prior information in higher education admissions, placement, and academic early-warning systems. Because they record academic performance before college entry, they can predict subsequent achievement to some extent (Richardson et al., 2012; Schneider and Preckel, 2017). Statistical prediction, however, does not imply developmental determination. Treating admission scores as stable ability labels overlooks the continuing interplay among motivation, engagement, and the educational environment after enrollment. This issue is especially salient in vocational higher education: students enter through multiple admission pathways whose institutional contexts and score comparability differ, yet they subsequently face common demands in vocational coursework, practical training, and occupational competence. The educationally and psychologically meaningful question is therefore not only whether admission scores predict vocational course achievement, but also whether malleable post-enrollment learning processes weaken that predictive association.

Learning engagement provides a critical entry point for this question. It captures students' behavioral, emotional, and cognitive connection with learning and reflects the energy, effort, persistence, and concentration invested in academic tasks (Fredricks et al., 2004; Kahu, 2013). Engagement also emerges at the interface between students' psychological resources and their educational environment (Kahu and Nelson, 2018). Existing work has primarily examined whether engagement is associated with average achievement. The present study instead tests whether engagement changes the strength of the association between prior academic standing and later-recorded course achievement. In resilience terminology, such an interaction is a protective model rather than a distinct new form of moderation (Fergus and Zimmerman, 2005).

The development of learning engagement is closely tied to students' judgments of their learning capabilities. Social cognitive theory proposes that self-efficacy is associated with goal selection, effort, persistence, and environmental regulation (Bandura, 1986; 1997; Schunk and DiBenedetto, 2020). In the present data, however, academic self-efficacy and engagement were measured concurrently at T1. Their association with later-extracted academic records therefore supports a theoretically consistent, partially time-ordered statistical pathway, not longitudinal causal mediation.

Whether individual engagement translates into academic outcomes also depends on the instructional context in which learning occurs. Teachers' respect for student autonomy, clear communication of course structure, effective guidance when difficulties arise, and relational support can help students direct their effort toward productive learning activities (Jang et al., 2010; Ryan and Deci, 2020). The educational-interface framework of student engagement similarly emphasizes that student factors and institutional contexts jointly shape engagement and its outcomes (Kahu and Nelson, 2018), and meta-analytic evidence indicates stable associations of positive teacher–student relationships with student engagement and academic achievement (Roorda et al., 2011). Recent studies of teacher caring, self-efficacy, and learning engagement have mainly examined how teacher support raises students' psychological resources and engagement (Du et al., 2026). Whether teacher support additionally improves the efficiency with which existing engagement is converted into objective achievement remains to be tested.

Career adaptability comprises concern, control, curiosity, and confidence resources used to manage vocational tasks and transitions (Savickas and Porfeli, 2012). Academic self-efficacy, engagement, and career adaptability were all measured at T1. H3 is therefore a cross-sectional indirect association only; reverse ordering, stable response tendencies, and unmeasured factors such as personality or prior career planning remain plausible alternatives.

The present study combines T0 admission records, a T1 survey, and same-semester course grades extracted after the survey period. Its primary contribution is a multisource test of whether learning engagement acts as a protective moderator of the admission-score–achievement association in vocational higher education. Secondary analyses examine cross-sectional indirect associations involving self-efficacy and engagement, and whether perceived teacher support modestly changes the engagement–achievement association. These aims were specified before inferential testing, but the study was not publicly preregistered.

## 2 Theoretical background and hypotheses

### 2.1 Social cognitive theory, learning engagement, and protective moderation

Social cognitive theory emphasizes reciprocal interplay among personal factors, behavior, and the environment (Bandura, 1986). Academic self-efficacy represents a capability belief, learning engagement a self-reported learning process, teacher support a perceived contextual resource, and vocational course achievement an administrative outcome. These theoretical roles do not imply that the concurrent T1 variables were causally ordered in the present observational design.

Resilience research distinguishes compensatory and protective models: a compensatory factor has an independent promotive association with the outcome, whereas a protective factor changes the risk–outcome association (Fergus and Zimmerman, 2005). The focal interaction in this study is therefore described as protective moderation. A lower standardized admission score is treated as a relative indicator of weaker prior academic standing within a comparable admission group, not as a stable risk identity.

### 2.2 Protective moderation of the admission score–achievement association by learning engagement

Two competing patterns are plausible. A cumulative-advantage or “rich-get-richer” account predicts that highly engaged students with stronger admission foundations use learning strategies and feedback more efficiently, thereby amplifying initial differences. A protective account instead predicts that sustained effort, repeated practice, feedback use, and strategy revision provide students with weaker prior foundations more opportunities to close knowledge gaps, reducing the slope of admission scores. H1 was specified a priori in the protective direction because engagement directly increases exposure to these corrective learning processes.

The protective hypothesis concerns a difference in slopes, not the elimination of admission-score differences. Under low engagement, current achievement may more strongly reproduce prior academic standing. Under high engagement, repeated practice and strategy revision may reduce that dependence. The empirical test therefore gives primary weight to the interaction coefficient, its confidence interval, and incremental explained variance; a non-significant simple slope at one moderator value is not treated as proof of a zero association.

H1: Learning engagement negatively moderates the association between standardized admission scores and vocational course achievement, consistent with a protective model in which the positive association becomes weaker as engagement increases.

### 2.3 Academic self-efficacy, learning engagement, and vocational course achievement

Academic self-efficacy refers to students' judgments of their capability to organize and execute the actions required to complete specific learning tasks. These judgments influence task choice, effort, persistence, and self-regulation (Bandura, 1997; Schunk and DiBenedetto, 2020; Honicke and Broadbent, 2016). In vocational courses, students with stronger self-efficacy are more likely to interpret challenges as manageable tasks and consequently to initiate, invest in, and persist with learning. Students with lower self-efficacy may instead construe difficulty as evidence of insufficient ability and reduce engagement or withdraw prematurely.

Learning engagement is examined as a statistical channel linking capability beliefs to a later-recorded objective outcome. Because academic self-efficacy and engagement were measured together at T1, the analysis does not establish that self-efficacy preceded engagement. Reverse association, reciprocal influence, prior study habits, and common response tendencies are plausible alternatives.

H2: Academic self-efficacy has a positive cross-sectional indirect association with later-recorded vocational course achievement through learning engagement, after admission scores are controlled.

### 2.4 Academic self-efficacy, learning engagement, and career adaptability

Career construction theory defines career adaptability as a psychosocial resource for managing current and anticipated vocational development tasks, transitions, and uncertainty. It encompasses career concern, career control, career curiosity, and career confidence (Savickas and Porfeli, 2012). Although these resources are future oriented, their development is not detached from students' present learning experiences. Through vocational courses, practical projects, and occupational qualification preparation, vocational college students gradually learn about occupational requirements, explore job pathways, and test their capability to perform work-related tasks. Social cognitive career theory likewise emphasizes that capability beliefs are closely related to academic and career interests, choices, and performance (Lent et al., 1994).

Academic self-efficacy may be associated with career adaptability partly because students who feel capable also report greater participation in vocational learning. However, career planning may likewise reinforce engagement and self-efficacy, and stable individual differences may generate all three reports. The model therefore tests covariance consistent with the proposed pathway rather than temporal mediation.

H3: Academic self-efficacy has a positive cross-sectional indirect association with career adaptability through learning engagement.

### 2.5 The moderating role of teacher support

Teacher support includes not only emotional care but also respect for student autonomy, clear communication of course structure, and effective guidance when students encounter difficulty. Self-determination theory proposes that autonomy support, structure, and relatedness support satisfy students' needs for autonomy, competence, and relatedness, thereby improving motivation and engagement (Ryan and Deci, 2020). The educational-interface framework of student engagement likewise emphasizes that institutional and student conditions interact at the learning interface (Kahu and Nelson, 2018). Rather than examining only whether teacher support increases engagement, the present study asks whether it changes the efficiency with which existing engagement is translated into objective achievement.

Vocational courses—particularly practical, project-based, and skills-oriented courses—often involve explicit task structures and intensive feedback. High teacher support can help students identify priorities, understand standards, correct errors, and connect vocational knowledge with work tasks, thereby directing their engagement more effectively toward course outcomes. Under low teacher support, even substantial engagement may not yield corresponding achievement when direction is unclear or feedback is insufficient. Recent research has linked teacher caring with self-efficacy, engagement, and perceived learning gains (Du et al., 2026); the present study positions teacher support as a contextual boundary on the engagement–objective achievement association.

H4: Teacher support positively moderates the association between learning engagement and vocational course achievement, such that the positive prediction of achievement by engagement becomes stronger at higher levels of teacher support.

### 2.6 Integrated research model

Figure 1 states the distinct statistical roles explicitly: engagement moderates the admission-score association in H1, serves as an intervening variable in the cross-sectional indirect tests H2 and H3, and is the focal predictor whose association with achievement is moderated by teacher support in H4. The roles are theoretically compatible because a construct may function as a process variable in one conditional equation and as a boundary condition in another; causal ordering is not inferred from this role assignment.

**Figure 1:**
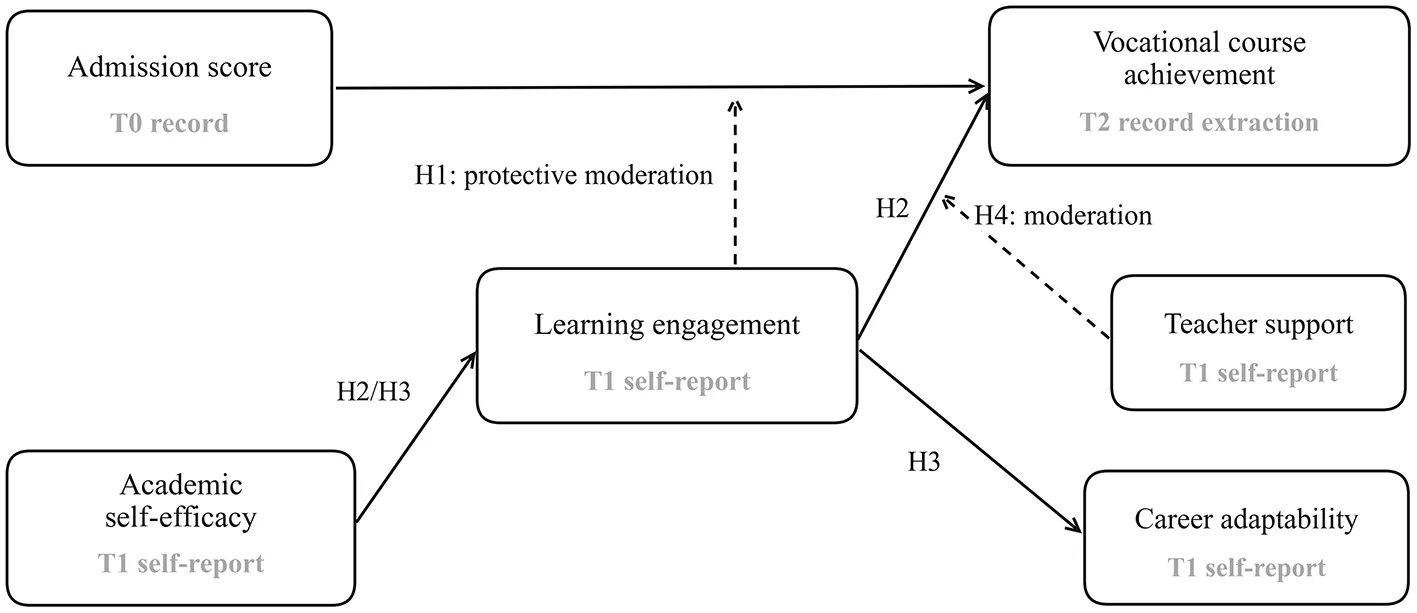
Integrated research model. Solid arrows represent focal associations; dashed arrows indicate moderation of the labeled association. Engagement is a moderator in H1, an intervening variable in the cross-sectional indirect tests H2 and H3, and a predictor whose achievement association is moderated in H4. T2 denotes the July 13, 2026 administrative extraction of same-semester grades, not a fully non-overlapping prospective outcome period.

## 3 Materials and methods

### 3.1 Study design

This study used a multisource observational design with partial temporal ordering. T0 admission scores were recorded when students entered college. T1 questionnaire data were collected from May 29 to June 29, 2026. Same-semester vocational course grades were extracted from the academic records system on July 13, 2026. Depending on each student's survey date, the interval from questionnaire completion to grade extraction was 14–45 days. The extraction occurred after the survey, but some graded learning activities may have overlapped the T1 collection window; “T2” denotes the administrative extraction point and does not establish a fully prospective outcome period.

The study was not a repeated-wave longitudinal panel. Academic self-efficacy, learning engagement, teacher support, and career adaptability were measured once at T1. H2 is partially time ordered only in the limited sense that the self-reports were collected before the administrative extraction of the achievement file, whereas H3 is fully cross-sectional. The four hypotheses were specified from the theoretical model before formal inferential analyses, but no public preregistration or time-stamped analysis plan was filed. Results should therefore be interpreted as theory-guided observational tests requiring independent confirmation.

### 3.2 Participants and procedure

The protocol was approved by the ethics review body of the researchers' institution (approval no. HZY20260525). The first questionnaire page described the purpose, data linkage, voluntary nature of participation, confidentiality, right to stop before submission, and research use of the records. Students proceeded only after indicating informed consent. Student identification numbers were used solely for deterministic linkage and were replaced with anonymous research codes after matching; access to the linkage file was restricted.

A total of 520 questionnaire records were obtained. Data cleaning sequentially excluded 18 records with invalid student identification numbers or records that could not be matched reliably, six records from special admission pathways such as the “3+2” pathway that lacked comparable admission scores, and five records without valid vocational course grades. The resulting analytical sample comprised 491 students, for a retention rate of 94.4%. Before formal analysis, duplicate identifiers, scale missingness, extreme response patterns, grade ranges, and standardization groups were rechecked. No additional records met the exclusion criteria; therefore, all 491 students were retained in the final analytical sample. Participant characteristics are summarized in Table 1.

**Table 1:** Demographic and learning-experience characteristics of the sample (N = 491).

Variable	Category	*n*	%
Sex	Male	221	45.0
	Female	270	55.0
Year of study	First year	78	15.9
	Second year	194	39.5
	Third year	219	44.6
Admission pathway	National College Entrance Examination	176	35.8
	Spring Academic Proficiency Test	174	35.4
	3+Certificate	141	28.7
Internship/practicum/enterprise-based learning	No	232	47.3
	Yes	259	52.7
Major-related occupational or skill-level certificate	Not obtained	279	56.8
	Obtained	212	43.2

The final sample included 221 men (45.0%) and 270 women (55.0%). Seventy-eight students were in their first year (15.9%), 194 in their second year (39.5%), and 219 in their third year (44.6%). The sample covered 11 academic majors. Regarding admission pathways, 176 students entered through the National College Entrance Examination (35.8%), 174 through the Spring Academic Proficiency Test (35.4%), and 141 through the “3+Certificate” pathway (28.7%). In addition, 259 students had completed an internship, practicum, or enterprise-based learning experience (52.7%), whereas 232 had not (47.3%). A total of 212 students had obtained an occupational qualification or skill-level certificate related to their major (43.2%), while 279 had not (56.8%).

### 3.3 Measures

#### 3.3.1 Admission scores

Admission scores were obtained from the college admissions management system. Because raw scores from different admission years, examination pathways, and scoring systems differ in test difficulty, maximum possible score, and score distribution, they are not directly comparable across groups. We therefore defined reference groups by admission year and comparable examination-scoring system. The National College Entrance Examination, Spring Academic Proficiency Test, and “3+Certificate” examination formed distinct scoring systems; when different examination categories or maximum scores remained within an admission pathway, reference groups were subdivided further. Raw admission scores were standardized within each reference group. The standardized admission score for student i in reference group g was calculated as follows:


$$\begin{array}{l} ADM_{-}Z_{ig}=\frac{X_{ig}-{\bar{X}}_{g}}{S_{g}} \end{array}$$

where ADM_RAW_*ig*_ is student i's raw admission score in reference group g, and M_g and SD_g are the mean and standard deviation of admission scores in that reference group, respectively. A value above 0 indicates that a student's admission score exceeded the mean of the comparable group, whereas a value below 0 indicates a score below that mean; the absolute value indicates the distance from the group mean in standard deviation units. Raw admission scores were used only for data verification and descriptive statistics. Standardized admission scores (ADM_Z) were used in correlations, regressions, and hypothesis tests.

#### 3.3.2 Academic self-efficacy

Academic self-efficacy was measured at T1. We drew on Chemers et al.'s (2001) assessment of college academic self-efficacy and on content related to self-efficacy for learning and performance and self-regulated learning in the Motivated Strategies for Learning Questionnaire (MSLQ; Pintrich et al., 1991). Eight items were adapted to the context of vocational college coursework. The MSLQ is designed to assess college students' course-related motivation and learning strategies and covers self-efficacy for learning and performance, cognitive and metacognitive strategies, and management of learning resources.

The eight items covered two domains: vocational course learning efficacy and self-regulated learning efficacy. The first four items assessed students' beliefs in their capability to understand vocational course content, complete learning tasks, and solve learning difficulties. The remaining four assessed beliefs in their capability to follow learning plans, persist, manage study time, and use learning resources proactively. Example items were “Even when a vocational course is difficult, I believe that I can learn the material” and “Even when I have many learning tasks, I can manage my time and complete them.”

All items were rated on a 5-point Likert scale from 1 (strongly disagree) to 5 (strongly agree), with no reverse-scored items. Mean scores were computed for each domain, and the mean of all eight items represented overall academic self-efficacy. Higher scores indicated stronger efficacy beliefs regarding vocational course learning and self-regulated learning tasks.

#### 3.3.3 Learning engagement

Learning engagement was measured at T1 with nine positively worded items organized as vigor, dedication, and absorption. The content was informed by the UWES-S structure (Schaufeli et al., 2002, 2006) but rewritten for vocational coursework, practical training, and project work. Because the administered items and agreement response format differ from the original frequency-based UWES, the measure is described as a context-adapted item set rather than the original UWES-S.

The adapted measure comprised three items for each dimension. Vigor captured students' energy, effort, and persistence when facing difficulty in vocational learning. Dedication captured meaning, interest, pride, and willingness to invest in vocational learning. Absorption captured sustained attention and immersion in vocational coursework, practical training, or projects. Example items were “I am willing to invest substantial effort when facing vocational learning tasks” and “I can become immersed in vocational coursework, practical training, or project-based learning.”

All nine items used a 5-point agreement scale from 1 (strongly disagree) to 5 (strongly agree). This common format was chosen to reduce switching across questionnaire sections, but it changes the response process from frequency to endorsement and may affect construct comparability. No separate equivalence study was conducted. Vigor, dedication, and absorption means and an overall nine-item mean were calculated; results therefore apply to the administered vocational-context agreement measure.

#### 3.3.4 Career adaptability

Career adaptability was measured at T1 with 12 context-adapted statements representing concern, control, curiosity, and confidence (Savickas and Porfeli, 2012; Maggiori et al., 2017; Rudolph et al., 2017). The original capability-strength response task was replaced by agreement ratings. Accordingly, the scores reflect endorsement of adapted career-adaptability statements and should not be assumed metrically equivalent to the original CAAS-SF.

The measure comprised 12 items, with three items for each dimension. Career concern reflected thinking about, attending to, and preparing for future career development. Career control reflected taking responsibility for one's career development, making proactive career decisions, and working toward career goals. Career curiosity reflected willingness to explore occupations, industries, career pathways, and career experiences. Career confidence reflected beliefs in one's capability to cope with job-search difficulties, perform basic occupational tasks, and adapt to changing work environments. Example items were “I proactively think about the direction of my future career” and “Even if the future work environment changes, I am confident that I can adapt.”

Items were rated from 1 (strongly disagree) to 5 (strongly agree). Dimension and overall means were computed from the responses actually administered. The agreement format facilitated a common questionnaire structure but limits direct comparison of raw means with studies using the original capability-strength scale.

#### 3.3.5 Teacher support

Teacher support was measured at T1 with nine context-specific items written with reference to autonomy support, structure, and teacher involvement in the Teacher as Social Context framework (Belmont et al., 1988; Skinner and Belmont, 1993). The item set is not presented as a validated standard short form of the TASCQ. The involvement content was labeled relatedness support because the administered statements focused on concern for learning difficulties, progress, and development.

The measure contained three items per dimension. Autonomy support reflected teachers' respect for students' ideas and choices in learning and their encouragement of independent thinking and problem solving. Structure support reflected whether teachers clearly communicated course objectives and learning requirements, provided concrete guidance when students encountered difficulty, and helped students understand the connection between vocational knowledge and work practice. Relatedness support reflected teachers' care, encouragement, and attention to students' learning difficulties, progress, and development. Example items were “Teachers respect my ideas and choices in vocational learning” and “When I encounter learning difficulties, teachers provide effective guidance.”

The nine positive statements used a 5-point agreement scale, rather than the original extent-of-fit format. Three-item means represented autonomy, structure, and relatedness support, and the nine-item mean represented overall perceived support. No independent expert content-validity review, cognitive interview, pilot test, or criterion-validity study was conducted before administration. The present evidence is therefore limited to internal structure, reliability, and associations in this sample.

All four self-report measures were administered in Chinese using the same 1–5 agreement scale. The contextual and response-format adaptations were made for questionnaire consistency and vocational relevance, not on the basis of demonstrated measurement equivalence. No separate expert review or pilot sample was available. We therefore report reliability, dimension-level CFA, item-level sensitivity CFA, Fornell–Larcker and HTMT evidence, and subgroup configural checks, while treating the measures as sample-specific and acknowledging that AVE below 0.50 and incomplete invariance evidence constrain strong construct claims. The exact administered Chinese wording and checked English translations are provided in Supplementary Table S1b.

#### 3.3.6 Vocational course achievement

Vocational course grades were extracted on July 13, 2026. The 491 students contributed 2,488 valid student–course records across 66 course codes. Each student contributed 4–6 courses (*M* = 5.07, SD = 0.74): 117 students had four courses, 224 had five, and 150 had six. A credit-weighted mean was calculated as raw vocational course achievement (PROF_RAW).


$$\begin{array}{l} PROF_{-}RAW_{i}=\frac{\overset{k}{\underset{j=1}{\sum }}Score_{ij}\times Credit_{ij}}{\overset{k}{\underset{j=1}{\sum }}Credit_{ij}} \end{array}$$

The primary composite did not directly model instructor identity because the administrative extract contained course codes but no instructor identifier. To address course composition and grading-level differences, sensitivity analyses (a) controlled course count, (b) standardized grades within course code before credit-weighted student aggregation, and (c) estimated student–course regressions with 66 course fixed effects and student-clustered standard errors. Course fixed effects absorb average grading and difficulty differences across course codes but cannot separate instructors teaching the same code.

Because curricula, assessment requirements, and progression through the training plan can differ across majors and admission cohorts, raw vocational course achievement is not directly comparable across groups. Reference groups were therefore defined by admission year and academic major, and PROF_RAW was standardized within each group to create standardized vocational course achievement (PROF_Z):


$$\begin{array}{l} PROF_{-}Z_{ig}=\frac{PROF_{-}RAW_{ig}-{\bar{PROF_{-}RAW}}_{g}}{SD\left(PROF_{-}RAW_{g}\right)} \end{array}$$

where PROF_RAW_*ig*_ is student i's raw vocational course achievement in reference group g, and M_g and SD_g are the mean and standard deviation of vocational course achievement in that reference group, respectively.

A PROF_Z value above 0 indicates achievement above the reference-group mean; a value below 0 indicates achievement below that mean. Its absolute value indicates the distance from the group mean in standard deviation units.

Descriptive statistics primarily report PROF_RAW to retain the intuitive meaning of course grades. Correlation analyses and hypothesis tests use PROF_Z to reduce incomparability associated with major and admission year. Robustness analyses reestimated the models with PROF_RAW replacing PROF_Z to determine whether the directions and statistical inferences of the focal effects remained consistent.

#### 3.3.7 Covariates

Covariates were selected on the basis of their theoretical relevance to the focal variables, temporal precedence, and data availability. The primary analyses controlled for sex, year of study, and admission pathway to account for demographic and admission-background differences. Sex was dummy coded with men as the reference group. Year of study and admission pathway were represented by dummy variables, with first-year students and National College Entrance Examination admission as the reference categories.

The role of standardized admission scores (ADM_Z) depended on the model. In models testing the association between admission scores and vocational course achievement, ADM_Z was the focal predictor. In models in which academic self-efficacy, learning engagement, teacher support, or career adaptability was the principal predictor, ADM_Z was included as a covariate to control students' academic foundations at college entry. Apart from the model-specific role of ADM_Z, the same covariate specification for sex, year of study, and admission pathway was used across the primary models.

Internship, practicum, or enterprise-based learning experience and possession of a major-related occupational qualification or skill-level certificate may influence academic performance and career development, but they may also result from students' prior learning processes and academic performance. To avoid overcontrol by potential post-exposure variables, these variables were excluded from the primary analyses and entered only as additional covariates in robustness analyses.

In robustness analyses using credit-weighted raw vocational course achievement (PROF_RAW) instead of PROF_Z, 10 dummy variables represented the 11 admission-year-by-major groups to control differences in curricula and grading standards. Because these groups already incorporated admission-year information, year-of-study dummy variables were not entered simultaneously, thereby avoiding redundant information and potential multicollinearity.

### 3.4 Data processing and analysis strategy

Primary analyses reproduced the prespecified SPSS/PROCESS models; additional revision analyses were conducted in Python using statsmodels and semopy. Data linkage, variable construction, descriptive analyses, robust regression, CFA, bootstrap inference, and course-level sensitivity analyses were scripted. Analytical code and construction specifications accompany the revision.

First, SPSS 27.0 was used to verify matches among questionnaire responses, admission scores, and vocational course grades and to check duplicate records, missing values, out-of-range values, and coding. Distributions of continuous variables were examined using means, standard deviations, skewness, kurtosis, and graphical displays. Multicollinearity, residual distributions, and potential heteroscedasticity were assessed before regression analysis. Cronbach's alpha evaluated the internal consistency of scales and dimensions. Categorical covariates were dummy coded according to prespecified reference groups: one dummy variable for sex and K-−1 dummy variables for year of study and admission pathway.

Measurement analyses first reproduced the prespecified dimension structures. To address the use of dimension-score parcels in the submitted model, an item-level sensitivity CFA estimated 12 correlated first-order factors from all 38 administered items, with no correlated residuals or *post hoc* item deletion. The dimension-score model was retained only as a parsimonious representation of the prespecified multidimensional instruments, not as the sole validity evidence.

Discriminant validity was evaluated using construct correlations, the Fornell–Larcker matrix (Fornell and Larcker, 1981), and heterotrait–monotrait ratios (HTMT; Henseler et al., 2015). Values below 0.85 were treated as conservative evidence that the four overall constructs were distinguishable. Item-level configural models were also estimated separately by sex and year of study; because equality constraints were not tested and the first-year subgroup was small (*n* = 78), these checks do not establish metric or scalar invariance.

Common method variance was examined with procedural controls, the single-factor benchmarks, and an unmeasured latent method-factor sensitivity model (Podsakoff et al., 2003). The latter added an orthogonal equal-loading random-intercept method factor to the 12-factor item-level measurement model. Fit change and H3 factor-score paths with and without the method factor were compared. The absence of a marker variable and the observational self-report design prevent method variance from being ruled out.

After evaluating the measurement models, means, standard deviations, and Pearson correlations were calculated in SPSS 27.0. Group-standardized vocational course achievement (PROF_Z) was the primary achievement outcome for hypothesis testing. The primary models controlled for sex, year of study, and admission pathway. Models in which academic self-efficacy, learning engagement, teacher support, or career adaptability served as a principal predictor additionally controlled for ADM_Z. When testing the admission score–achievement association, ADM_Z was the focal predictor and was not entered again as a covariate.

H1 and H4 were tested with mean-centered product terms and HC3 standard errors. Centering makes each constituent main effect conditional on the other interacting variable being at its sample mean; it does not alter the interaction coefficient or model fit. Interactions were summarized with unstandardized and fully standardized coefficients, bootstrap confidence intervals, ΔR^2^, simple slopes, and Johnson–Neyman regions. The number and proportion of observations in the regions were reported.

H2 and H3 used 5,000 case-resampling bootstrap draws. Both unstandardized and completely standardized indirect effects received bootstrap standard errors and 95% percentile intervals. The models estimate statistical indirect associations; concurrent measurement of self-efficacy and engagement precludes temporal mediation claims.

A non-central-F sensitivity analysis quantified the detectable incremental effect at *N* = 491 and α = 0.05. An integrated model then included academic self-efficacy, admission score, engagement, teacher support, both focal interactions (admission score × engagement and engagement × teacher support), and the baseline covariates in the achievement equation; the engagement equation was regressed on self-efficacy and covariates. Conditional indirect associations were bootstrapped at teacher support −1 SD, the mean, and +1 SD.

Robustness analyses added internship and certification covariates, used raw grades, controlled course count, created a credit-weighted outcome from grades standardized within course code, estimated course fixed-effects regressions with student-clustered standard errors, and examined H1 separately by admission pathway. A joint robust Wald test evaluated whether the interaction differed across pathways.

## 4 Results

### 4.1 Data screening

Following the matching and screening criteria described above, the 520 questionnaire records and corresponding admissions and academic records were verified. Eighteen records with invalid identifiers or unreliable matches were excluded, followed by six records from special admission pathways that lacked comparable admission scores and five records without valid vocational course grades. The final sample comprised 491 students, yielding a retention rate of 94.4%.

The final analytical dataset contained no missing values for the 38 self-report items or the principal study variables; therefore, no missing-data imputation was performed. All scale responses fell within the intended range of 1–5. No duplicate research codes or anomalous codes were identified. Skewness for the six principal continuous variables ranged from −0.25 to 0.15 and kurtosis from −0.36 to −0.06, indicating no marked departures from normality.

Outlier screening identified one raw vocational course score with an overall standardized value below −3.29. Verification against the original academic record showed no data-entry error, and the observation did not meet the extreme-value criterion after standardization within its admission-year-by-major reference group. It was therefore retained. All subsequent analyses used complete data from 491 students.

### 4.2 Measurement models, reliability, and validity

The prespecified dimension-level CFA models fit well, but the excellent fit of the 12-indicator overall model partly reflects the use of prespecified dimension scores as parcels. An item-level sensitivity model with 12 correlated first-order factors and all 38 items also fit well without correlated errors or item deletion, χ^2^(599) = 595.855, CFI = 1.000, TLI = 1.000, RMSEA = 0.000. Standardized loadings in the more restrictive four-overall-factor item model ranged from 0.512 to 0.647. These sample-specific results support the administered dimensional structure but do not substitute for cross-validation.

The original dimension-score four-construct model and its competing models are retained in Table 2 for comparability with the submitted analysis. Because the item-level multidimensional model directly represents the item structure, substantive interpretation now relies on both analyses rather than on the unusually high parcel-model fit alone. Reliability and convergent-validity results are reported in Table 3.

**Table 2:** Comparison of measurement and competing models (N = 491).

Measure	Model	χ^2^	*df*	χ^2^/*df*	CFI	TLI	RMSEA	RMSEA 90% CI	AIC	BIC
Academic self-efficacy	One-factor model	215.11	20	10.755	0.823	0.752	0.141	[0.124, 0.158]	247.11	314.253
	Correlated two-factor model	21.675	19	1.141	0.998	0.996	0.017	[0.000, 0.044]	55.675	127.015
Learning engagement	One-factor model	250.644	27	9.283	0.808	0.744	0.130	[0.116, 0.145]	286.644	362.18
	Correlated three-factor model	38.051	24	1.585	0.988	0.982	0.035	[0.010, 0.054]	80.051	168.176
Career adaptability	One-factor model	291.574	54	5.400	0.857	0.825	0.095	[0.084, 0.106]	339.574	440.289
	Correlated four-factor model	41.73	48	0.869	1.000	1.000	0.000	[0.000, 0.023]	101.73	227.623
	Second-order four-dimension model	42.085	50	0.842	1.000	1.000	0.000	[0.000, 0.020]	98.085	215.585
Teacher support	One-factor model	237.297	27	8.789	0.814	0.752	0.126	[0.112, 0.141]	273.297	348.833
	Correlated three-factor model	35.977	24	1.499	0.989	0.984	0.032	[0.000, 0.052]	77.977	166.102
Overall measurement model	Theoretical four-factor model	31.391	48	0.654	1.000	1.000	0.000	[0.000, 0.000]	91.391	217.285
	Three-factor model combining ASE and LE	67.585	51	1.325	0.988	0.985	0.026	[0.000, 0.041]	121.585	234.889
	Overall one-factor model	474.823	54	8.793	0.699	0.632	0.126	[0.116, 0.137]	522.823	623.538

ASE, academic self-efficacy; LE, learning engagement; CFI, comparative fit index; TLI, Tucker–Lewis index; RMSEA, root mean square error of approximation; AIC, Akaike information criterion; BIC, Bayesian information criterion.

**Table 3:** Internal consistency and convergent validity of the measures (N = 491).

Construct and dimension	Items	Cronbach's α	CR	AVE
Academic self-efficacy	8	0.817	—	—
Vocational course learning efficacy	4	0.783	0.784	0.476
Self-regulated learning efficacy	4	0.770	0.770	0.456
Learning engagement	9	0.817	—	—
Vigor	3	0.747	0.746	0.495
Dedication	3	0.722	0.725	0.467
Absorption	3	0.712	0.710	0.449
Career adaptability	12	0.856	—	—
Career concern	3	0.721	0.721	0.464
Career control	3	0.716	0.717	0.458
Career curiosity	3	0.706	0.706	0.445
Career confidence	3	0.753	0.753	0.505
Second-order career adaptability factor	Four first-order dimensions	—	0.881	0.649
Teacher support	9	0.813	—	—
Autonomy support	3	0.730	0.728	0.472
Structure support	3	0.720	0.717	0.459
Relatedness support	3	0.709	0.714	0.456

CR, composite reliability; AVE, average variance extracted.

Total-scale reliability ranged from 0.813 to 0.856. Overall-construct CR values from the item-level four-factor sensitivity model ranged from 0.814 to 0.856, whereas AVE ranged from 0.327 to 0.360; first-order AVE in the prespecified dimension models ranged from 0.445 to 0.505. Thus, internal consistency was acceptable but convergent validity remained weaker than ideal. In the Fornell–Larcker matrix, each square root of AVE (0.572–0.600) exceeded the corresponding observed construct correlations (largest |*r*| = 0.494). HTMT values ranged from 0.134 to 0.604, all below 0.85. The evidence supports discriminant validity more strongly than convergent validity; accordingly, associations involving these adapted measures should be interpreted as sample-specific rather than as evidence that their measurement properties have been fully established across populations.

### 4.3 Common method bias

Common method bias was evaluated through procedural and statistical approaches. Procedurally, participants were informed that there were no right or wrong answers and that collected information would be kept confidential; student identification numbers were used only for matching and removed immediately afterward. Academic self-efficacy, learning engagement, career adaptability, and teacher support were self-reported, whereas admission scores and vocational course grades came from the admissions and academic records systems, respectively. This multisource design reduced the potential for systematic bias attributable to a single data source.

The item-level 12-factor model with an orthogonal equal-loading method factor produced χ^2^(598) = 592.452, *CFI* = 1.000, *TLI* = 1.000, and *RMSEA* = 0.000. Relative to the trait-only item model, the fit change was negligible (Δ*CFI* < 0.001; Δ*RMSEA* = 0.000) and AIC/BIC increased. Factor-score sensitivity estimates for H3 were similar: the indirect-product estimate was 0.271 without and 0.284 with the method factor. These results do not demonstrate absence of method bias, but they indicate that the H3 pattern was not attenuated by this particular method-factor specification. H3 remains fully cross-sectional and self-reported and is interpreted conservatively; this method-factor result is a sensitivity check, not evidence that common method variance has been eliminated.

### 4.4 Descriptive statistics and correlations

Table 4 presents means, standard deviations, and Pearson correlations for the principal variables. The credit-weighted raw mean for vocational courses was 82.67 (*SD* = 5.90), with an observed range of 62.90–96.50. Mean scores for academic self-efficacy, learning engagement, career adaptability, and teacher support were 3.53 (*SD* = 0.58), 3.57 (*SD* = 0.55), 3.60 (*SD* = 0.54), and 3.60 (*SD* = 0.54), respectively.

**Table 4:** Descriptive statistics and correlations among the principal variables (N = 491).

Variable	*M*	*SD*	1	2	3	4	5	6
1. Standardized admission score	0.00	0.99	1					
2. Academic self-efficacy	3.53	0.58	0.232^***^	1				
3. Learning engagement	3.57	0.55	0.017	0.494^***^	1			
4. Career adaptability	3.60	0.54	−0.050	0.335^***^	0.410^***^	1		
5. Teacher support	3.60	0.54	−0.023	0.103^*^	0.244^***^	0.279^***^	1	
6. Standardized vocational course achievement	−0.01	0.99	0.253^***^	0.289^***^	0.395^***^	0.186^***^	0.102^*^	1

^*^p < 0.05; ^***^p < 0.001. Standardized admission scores were computed separately within multiple admission-year × pathway × examination-category reference groups and then merged; because the pooled sample combines group-specific standardizations, rounding and group composition yield an overall *SD* of 0.99 rather than exactly 1.00.

Admission scores correlated positively with achievement (*r* = 0.253, *p* < 0.001) but not with engagement (*r* = 0.017, *p* = 0.707) or career adaptability (*r* = −0.050, *p* = 0.267). The near-zero admission–adaptability correlation is consistent with these variables indexing different domains—relative prior academic standing vs. perceived career-development resources—but it may also reflect mixed admission systems and suppression after covariate adjustment. No causal or deficit interpretation is attached to this non-significant correlation.

Absolute correlations among the principal variables ranged from 0.017 to 0.494, and no excessively high zero-order correlations were observed. These results describe preliminary bivariate associations but do not establish indirect or moderating effects. H1–H4 were therefore evaluated using the interaction terms, bootstrap indirect effects, and simple-slope results.

### 4.5 Protective moderation by learning engagement

PROCESS 4.2 Model 1 was used to test whether learning engagement moderated the association between admission scores and vocational course achievement, with HC3 heteroscedasticity-consistent standard errors (Hayes, 2022). The protective-moderation results are presented in Table 5. Sex, year of study, and admission pathway were controlled. The overall regression model was significant, *F*(8, 482) = 22.038, *p* < 0.001, *R*^2^ = 0.240, indicating that the predictors jointly explained approximately 24.0% of the variance in standardized vocational course achievement.

**Table 5:** Protective moderating effect of learning engagement (N = 491).

Predictor	*B*	*SE*	*t*	*p*	95% CI
Standardized admission score	0.244	0.039	6.190	< 0.001	[.166, 0.321]
Learning engagement	0.677	0.072	9.358	< 0.001	[.534, 0.819]
Admission score × Learning engagement	−0.263	0.068	−3.864	< 0.001	[−0.397, −0.129]
Overall model	F(8, 482) = 22.038	—	—	< 0.001	R^2^ = 0.240
Interaction increment	ΔR^2^ =0.021	ΔF(1, 482) = 14.927	—	< 0.001	Standardized interaction = −0.144, 95% bootstrap CI [−0.214, −0.071]

At the sample mean of learning engagement, standardized admission scores were positively associated with standardized vocational course achievement, *B* = 0.244, *SE* = 0.039, *t* = 6.190, *p* < 0.001, 95% CI [0.166, 0.321]. At the sample mean of standardized admission scores, learning engagement was also positively associated with standardized achievement, *B* = 0.677, *SE* = 0.072, *t* = 9.358, *p* < 0.001, 95% CI [0.534, 0.819].

The admission score × engagement interaction was negative, *B* = −0.263, HC3 *SE* = 0.068, *t* = −3.863, *p* < 0.001, 95% CI [−0.397, −0.129], ΔR^2^ = 0.021, ΔF(1, 482) = 14.927, *p* < 0.001. The fully standardized interaction was −0.144, 95% bootstrap CI [−0.214, −0.071]. The interaction accounted for a small but non-trivial increment beyond the main effects and covariates.

At low engagement (−1 SD), the admission-score slope was *B* = 0.388, 95% CI [0.286, 0.491]; at the mean it was *B* = 0.244, 95% CI [0.166, 0.321]; and at high engagement (+1 SD) it was *B* = 0.099, *p* = 0.078, 95% CI [−0.011, 0.210]. The high-engagement interval still permits a small-to-moderate positive association, so non-significance is interpreted as statistical imprecision rather than evidence that the slope was zero.

As shown in Figure 2, the simple slope was steeper under low learning engagement and substantially flatter under high engagement. When engagement was low, vocational course achievement was more strongly linked to students' admission foundations; as engagement increased, achievement differences between students with different admission foundations narrowed.

**Figure 2:**
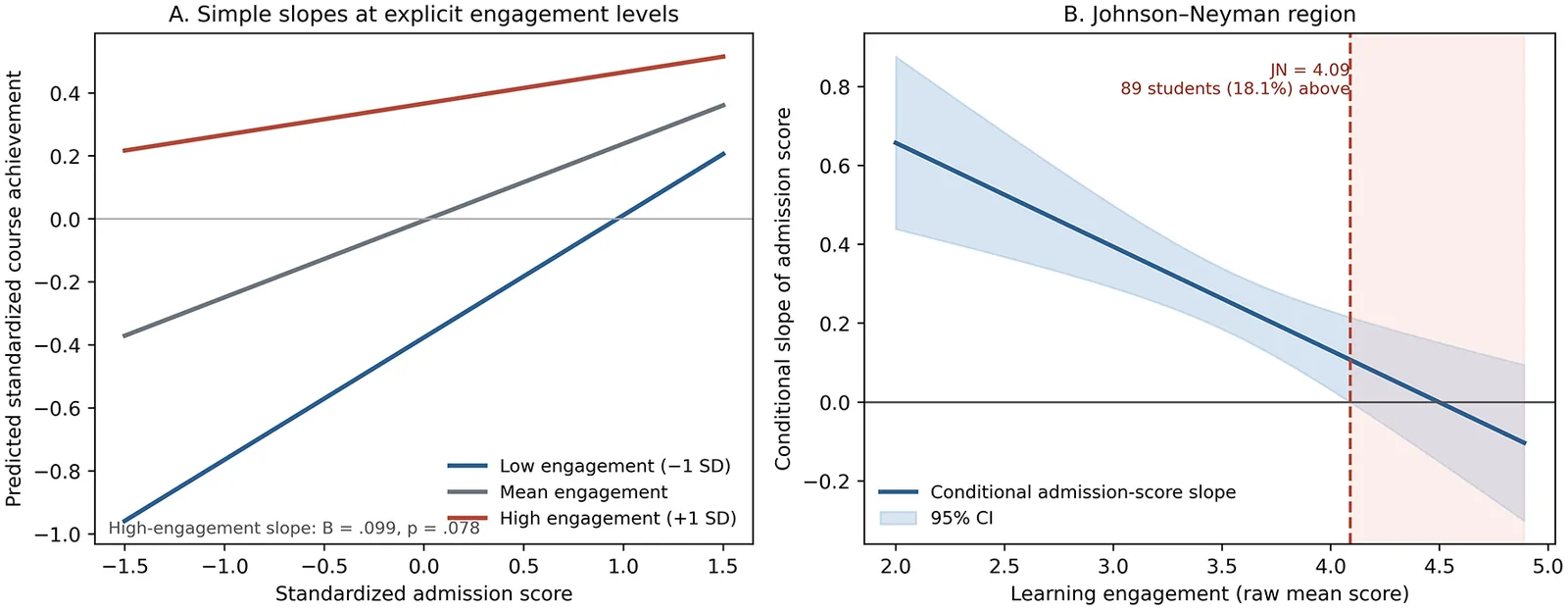
Learning engagement moderates the admission score–achievement association. Panel **(A)** shows predicted standardized achievement at engagement −1 *SD*, the mean, and +1 *SD*. Panel **(B)** shows the conditional admission-score slope and 95% confidence interval across the observed engagement range. The Johnson–Neyman threshold was 4.09; 89 students (18.1%) were above it.

The Johnson–Neyman threshold was a raw engagement score of 4.09. Eighty-nine students (18.1%) were above this value. Coverage in this region was therefore meaningful but smaller than in the significant-slope region, which reduced precision. The primary evidence for H1 is the negative interaction and slope decline, not the null-hypothesis test at high engagement.

Together, the interaction coefficient, standardized effect, ΔR^2^, conditional slopes, and coverage-adjusted Johnson–Neyman analysis supported a protective pattern. H1 was supported as a weakening, not an elimination, of the admission-score association.

### 4.6 Indirect association of academic self-efficacy with vocational course achievement through learning engagement

PROCESS 4.2 Model 4 was used to test the indirect association of academic self-efficacy with vocational course achievement through learning engagement. Confidence intervals were estimated with 5,000 bootstrap resamples. The model controlled for standardized admission scores, sex, year of study, and admission pathway, and regression coefficients used HC3 standard errors. Table 6 presents the results.

**Table 6:** Indirect effect of academic self-efficacy on vocational course achievement through learning engagement (N = 491).

Effect path	*B*/Effect	*SE*/BootSE	*t*	*p*	95% CI	Standardized effect
Academic self-efficacy → Learning engagement (a)	0.491	0.038	12.768	< 0.001	[0.415, 0.566]	0.519
Learning engagement → Vocational course achievement (b)	0.654	0.087	7.511	< 0.001	[0.483, 0.825]	0.362
Academic self-efficacy → Vocational course achievement: total effect (c)	0.419	0.079	5.302	< 0.001	[0.264, 0.574]	0.246
Academic self-efficacy → Vocational course achievement: direct effect (c′)	0.098	0.086	1.141	0.254	[−0.071, 0.267]	0.058
Academic self-efficacy → Learning engagement → Vocational course achievement (ab)	0.321	0.047	—	—	[0.232, 0.416]	0.188, BootSE = 0.027, 95% CI [.138, 0.241]

First, the regression model predicting learning engagement was significant, *F*(7, 483) = 24.626, *p* < 0.001, *R*^2^ = 0.258. Academic self-efficacy positively predicted engagement, *B* = 0.491, *SE* = 0.038, *t* = 12.768, *p* < 0.001, 95% CI [0.415, 0.566], indicating that students with stronger academic self-efficacy reported greater learning engagement.

Second, the model predicting standardized vocational course achievement was also significant, *F*(8, 482) = 16.768, *p* < 0.001, *R*^2^ = 0.222. With academic self-efficacy, learning engagement, and the covariates entered simultaneously, learning engagement positively predicted achievement, *B* = 0.654, *SE* = 0.087, *t* = 7.511, *p* < 0.001, 95% CI [0.483, 0.825]. The direct effect of academic self-efficacy was not significant, *B* = 0.098, *SE* = 0.086, *t* = 1.141, *p* = 0.254, 95% CI [−0.071, 0.267].

The 5,000-draw bootstrap indirect association through engagement was 0.321, BootSE = 0.047, 95% CI [0.232, 0.416]. The completely standardized indirect effect was 0.188, BootSE = 0.027, 95% CI [0.138, 0.241]. Because self-efficacy and engagement were concurrent, these estimates quantify an indirect association rather than temporal mediation.

Overall, stronger academic self-efficacy was associated with greater learning engagement, which in turn was associated with better vocational course achievement. After engagement was entered, the direct effect of self-efficacy was not significant, indicating that the association between academic self-efficacy and achievement in this model was primarily expressed through the statistical indirect pathway via engagement. Because academic self-efficacy and engagement were both measured at T1, the findings support the indirect association proposed in H2 but do not establish temporal ordering or causal mediation.

### 4.7 Indirect association of academic self-efficacy with career adaptability through learning engagement

H3 tested whether academic self-efficacy was indirectly associated with career adaptability through learning engagement. The indirect-effect results for career adaptability are presented in Table 7. Because all three constructs were measured at T1, the result is interpreted as a concurrent statistical indirect association rather than a temporally ordered causal process.

**Table 7:** Indirect effect of academic self-efficacy on career adaptability through learning engagement (N = 491).

Effect path	*B*/Effect	*SE*/Boot*SE*	*t*	*p*	95% CI	Standardized effect
Academic self-efficacy → Learning engagement (a)	0.491	0.038	12.768	< 0.001	[0.415,0.566]	0.519
Learning engagement → Career adaptability (b)	0.312	0.046	6.766	< 0.001	[0.222,0.403]	0.319
Academic self-efficacy → Career adaptability: total effect (c)	0.333	0.040	8.254	< 0.001	[0.254,0.412]	0.359
Academic self-efficacy → Career adaptability: direct effect (c′)	0.180	0.045	4.003	< 0.001	[0.091,0.268]	0.194
Academic self-efficacy → Learning engagement → Career adaptability (ab)	0.153	0.025	—	—	[0.106,0.202]	0.165, BootSE =0.025, 95% CI [0.117,0.217]

The regression model predicting learning engagement was significant, *F*(7, 483) = 24.626, *p* < 0.001, *R*^2^ = 0.258. Academic self-efficacy was positively associated with engagement, *B* = 0.491, *SE* = 0.038, *t* = 12.768, *p* < 0.001, 95% CI [0.415, 0.566], with a standardized coefficient of β = 0.519.

The regression model predicting career adaptability was also significant, *F*(8, 482) = 17.144, *p* < 0.001, *R*^2^ = 0.213. When academic self-efficacy, learning engagement, and the covariates were entered simultaneously, learning engagement was positively associated with career adaptability, *B* = 0.312, *SE* = 0.046, *t* = 6.766, *p* < 0.001, 95% CI [0.222, 0.403], β = 0.319. The direct association between academic self-efficacy and career adaptability remained significant, *B* = 0.180, *SE* = 0.045, *t* = 4.003, *p* < 0.001, 95% CI [0.091, 0.268], β = 0.194.

Without learning engagement in the outcome model, the total association between academic self-efficacy and career adaptability was significant, *B* = 0.333, *SE* = 0.040, *t* = 8.254, *p* < 0.001, 95% CI [0.254, 0.412], β = 0.359.

The 5,000-draw bootstrap indirect association was 0.153, BootSE = 0.025, 95% CI [0.106, 0.202]; the completely standardized estimate was 0.165, BootSE = 0.025, 95% CI [0.117, 0.217]. The direct association of self-efficacy with career adaptability remained B = 0.180, p < 0.001. These are cross-sectional associations and may reflect reverse or reciprocal relationships and shared response processes.

In summary, students with stronger academic self-efficacy generally reported greater learning engagement, which was in turn associated with stronger career adaptability. The direct association between academic self-efficacy and career adaptability remained significant after engagement was entered, indicating that engagement explained part, but not all, of their association. H3 was supported as a significant direct association and an additional indirect association through learning engagement.

### 4.8 Moderating role of teacher support

PROCESS 4.2 Model 1 was used to test whether teacher support moderated the association between learning engagement and vocational course achievement, using HC3 standard errors. The model controlled for standardized admission scores, sex, year of study, and admission pathway. The overall model was significant, *F*(9, 481) = 15.595, *p* < 0.001, *R*^2^ = 0.229, indicating that the predictors jointly explained 22.9% of the variance in standardized vocational course achievement.

At the sample mean of teacher support, learning engagement was positively associated with standardized vocational course achievement, *B* = 0.718, *SE* = 0.078, *t* = 9.165, *p* < 0.001, 95% CI [0.564, 0.872]. At the sample mean of engagement, the conditional association between teacher support and achievement was not significant, *B* = 0.027, *SE* = 0.081, *t* = 0.335, *p* = 0.738, 95% CI [−0.131, 0.186]. The nonsignificant conditional main effect of teacher support does not preclude interpretation of the interaction.

The engagement × teacher support interaction was positive, *B* = 0.348, HC3 *SE* = 0.155, *p* = 0.025, 95% CI [0.044, 0.651], Δ*R*^2^ = 0.009, Δ*F*(1, 481) = 5.044, *p* = 0.025. The fully standardized interaction was 0.104, 95% bootstrap CI [0.014, 0.195]. This is a small and comparatively imprecise interaction.

Conditional effects were then estimated at low, mean, and high teacher support. At low support (1 SD below the mean), learning engagement was positively associated with achievement, *B* = 0.530, *SE* = 0.108, *t* = 4.907, *p* < 0.001, 95% CI [0.318, 0.742]. At mean support, the association was *B* = 0.718, *SE* = 0.078, *t* = 9.165, *p* < 0.001, 95% CI [0.564, 0.872]. At high support (1 SD above the mean), the positive association was stronger, *B* = 0.907, *SE* = 0.121, *t* = 7.487, *p* < 0.001, 95% CI [0.669, 1.144].

Simple-slope analyses showed that learning engagement positively predicted vocational course achievement at low, mean, and high levels of teacher support, and that the slope increased with support (Table 8 and Figure 3). H4 was therefore supported.

**Table 8:** Moderating effect of teacher support (N = 491).

Predictor	*B*	*SE*	*t*	*p*	95% CI
Learning engagement	0.718	0.078	9.165	< 0.001	[0.564, 0.872]
Teacher support	0.027	0.081	0.335	0.738	[−0.131, 0.186]
Learning engagement × Teacher support	0.347	0.155	2.246	0.025	[0.044, 0.651]
Overall model	*F*(9, 481) = 15.595	—	—	< 0.001	*R^2^* = 0.229
Interaction increment	*ΔR^2^* =0.009	*ΔF*(1, 481) = 5.044	—	0.025	Standardized interaction = 0.104, 95% bootstrap CI [0.014, 0.195]

**Figure 3:**
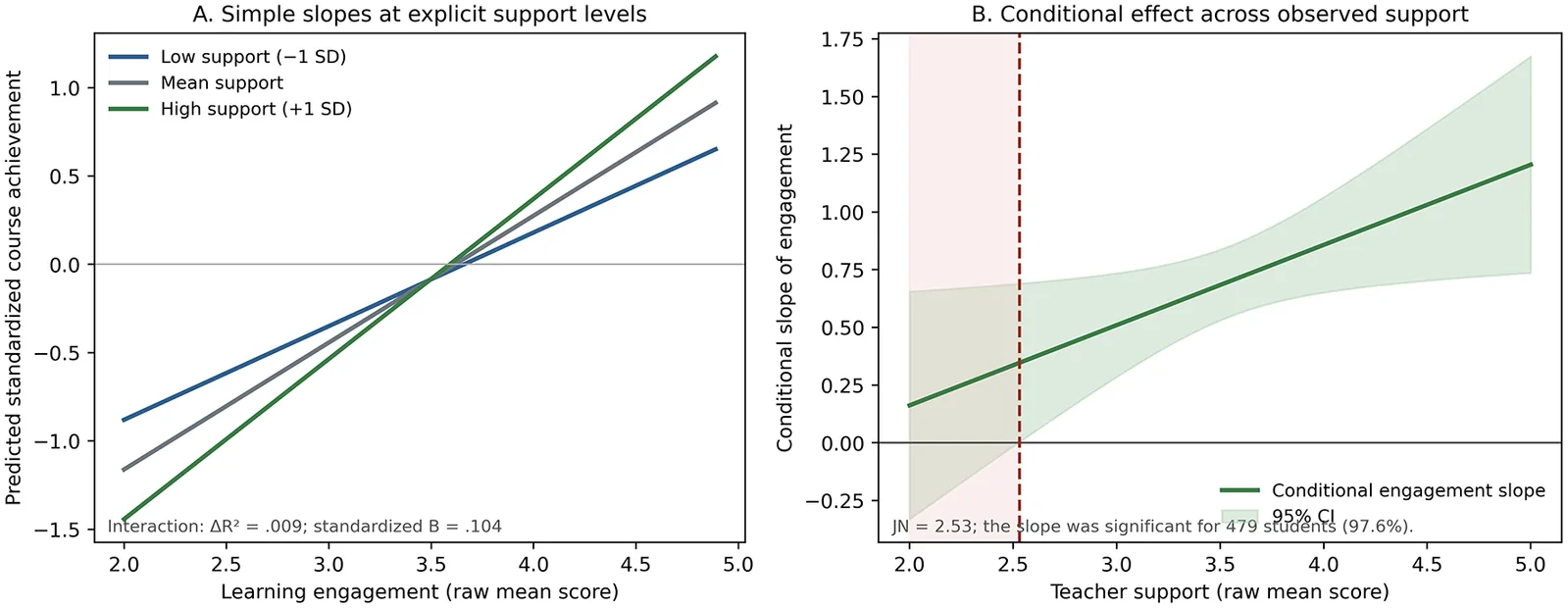
Teacher support moderates the engagement–achievement association. Panel **(A)** shows simple slopes at support −1 *SD*, the mean, and +1 *SD*. Panel **(B)** shows the conditional engagement slope and 95% confidence interval across observed support. The interaction was small (ΔR^2^ =0.009), and the conditional slope was statistically distinguishable from zero above support 2.53 (479 students; 97.6%).

The conditional engagement slope became statistically distinguishable from zero above teacher support 2.53; 479 students (97.6%) were at or above that value. H4 was supported in this sample, but the small Δ*R*^2^, *p* value near 0.05, and sensitivity results below warrant replication rather than a strong intervention claim.

### 4.9 Robustness analyses

The expanded sensitivity analyses addressed covariate choice, outcome construction, course composition, pathway heterogeneity, model integration, and interaction detectability. Detailed coefficients are reported in Supplementary Tables S4–S6. Robustness results are summarized in Table 9.

**Table 9:** Robustness tests of the principal findings (N = 491).

Hypothesis and focal effect	Primary model	Extended-covariate model	Alternative-outcome model	Conclusion
H1: Admission score × Learning engagement → Vocational course achievement	*B* = −0.263, *p* < 0.001, 95% CI [−0.397, −0.129]; *ΔR^2^* =0.021	*B* = −0.256, *p* < 0.001, 95% CI [−0.390, −0.121]; *ΔR^2^* = 0.019	*B* = −1.581, *p* < 0.001, 95% CI [−2.347, −0.815]; *ΔR^2^* =0.021	Robust
H2: Academic self-efficacy → Learning engagement → Vocational course achievement	*ab* =0.321, 95% bootstrap CI [0.232, 0.416]; *abcs* = 0.188	*ab* =0.321, 95% bootstrap CI [0.232, 0.416]; *abcs* = 0.188	*ab* = 1.820, 95% bootstrap CI [1.310, 2.380]; *abcs* = 0.179, 95% CI [0.131, 0.231]	Robust
H3: Academic self-efficacy → Learning engagement → Career adaptability	*ab* =0.153, 95% bootstrap CI [0.106, 0.202]; *abcs* = 0.165	*ab* =0.153, 95% bootstrap CI [0.105, 0.202]; *abcs* = 0.165	—	Robust
H4: Learning engagement × Teacher support → Vocational course achievement	*B* = 0.347, *p* = 0.025, 95% CI [0.044, 0.651]; *ΔR^2^* = 0.009	*B* = 0.337, *p* = 0.032, 95% CI [0.029, 0.645]; *ΔR^2^* = 0.008	*B* = 2.162, p = 0.017, 95% CI [0.390, 3.934]; ΔR^2^ = 0.009	Robust
Integrated model	H1: *B* = −0.265, *p* < 0.001	H4: *B* = 0.318, *p* = 0.030	Conditional indirect ab = 0.225/0.310/0.394; index = 0.156, 95% bootstrap CI [0.012, 0.291]	Consistent
Course-adjusted H1	Course fixed effects: *B* = −1.546, *p* < 0.001	Course-standardized aggregate: *B* = −0.267, *p* < 0.001	Course-count control: *B* = −0.263, *p* < 0.001	Robust
Pathway heterogeneity	NCEE: *B* = −0.293, *p* =0.025	Spring: *B* = −0.128, *p* = 0.315; 3+Certificate: *B* = −0.383, p = 0.001	Joint three-way test: *F*(2, 476) = 1.265, *p* =0.283	No detected heterogeneity

In the integrated model, the admission score × engagement interaction remained *B* = −0.265, HC3 *SE* = 0.067, *p* < 0.001, 95% CI [−0.396, −0.133], and the engagement × teacher support interaction remained *B* = 0.318, HC3 *SE* = 0.146, *p* = 0.030, 95% CI [.031, 0.605]. The direct self-efficacy coefficient was nonsignificant (*B* = 0.103, *p* = 0.235). Conditional indirect associations from self-efficacy through engagement to achievement were 0.225 (95% bootstrap CI [.123, 0.335]), 0.310 ([0.219, 0.402]), and 0.394 ([0.267, 0.525]) at teacher support −1 SD, the mean, and +1 SD, respectively. The index of moderated mediation was 0.156, BootSE = 0.070, 95% bootstrap CI [0.012, 0.291]. Thus, H1 survived the full equation, and the H2 indirect association increased modestly with teacher support.

Course-level evidence was consistent with H1. The 2,488 student–course observations covered 66 course codes. A course fixed-effects regression with student-clustered standard errors yielded an admission score × engagement coefficient of −1.546 raw grade points (*SE* = 0.377, *p* < 0.001, 95% CI [−2.287, −0.805]). When grades were first standardized within course code and then credit-weighted to the student level, the interaction was *B* = −0.267, *SE* = 0.069, *p* < 0.001. Controlling the number of courses produced *B* = −0.263, *SE* = 0.069, *p* < 0.001.

Pathway-stratified H1 estimates were negative for the National College Entrance Examination (*B* = −0.293, *p* = 0.025, *n* = 176), Spring Academic Proficiency Test (*B* = −0.128, *p* = 0.315, *n* = 174), and 3+Certificate pathway (*B* = −0.383, *p* = 0.001, *n* = 141). The two three-way interaction terms were not jointly significant, robust *F*(2, 476) = 1.265, *p* = 0.283. The pooled protective pattern was therefore not statistically heterogeneous across pathways, although the subgroup intervals were wide and pathway-specific score meanings remain a limitation.

A noncentral-*F* sensitivity analysis at *N* = 491 and α = 0.05 indicated minimum detectable incremental *f*^2^ values of 0.016 for 80% power and 0.021 for 90% power. H1 had *f*^2^ = 0.028 and estimated power 0.957; H4 had *f*^2^ = 0.012 and estimated power 0.666. These results support adequate sensitivity for H1 but limited sensitivity and greater replication uncertainty for H4.

The previously reported extended-covariate and raw-outcome models also remained directionally consistent. Across specifications, H1 was stable. H4 remained positive but small and less precisely estimated, so its practical interpretation has been reduced throughout the manuscript.

Overall, the new analyses strengthen the robustness of the protective H1 interaction while also sharpening the boundary of the claims: course and pathway adjustments did not explain the pattern, but the design remains observational, measurement evidence is sample-specific, and the H4 interaction is modest.

## 5 Discussion

### 5.1 Principal findings

The central result was a small protective interaction: admission scores were positively associated with same-semester achievement, and this association weakened as engagement increased. The high-engagement slope was statistically imprecise, not demonstrated to be zero. Cross-sectional indirect associations connected self-efficacy, engagement, achievement, and career adaptability, while teacher support showed a smaller moderation effect. Integrated, course-adjusted, and pathway analyses did not alter the H1 conclusion.

#### 5.1.1 Does learning engagement weaken the continuing influence of admission scores?

H1 is best understood as an application of the established protective model, not as a new moderation construct (Cohen, 1988). The negative interaction favors the protective account over the competing rich-get-richer prediction in this sample: admission-score differences were less strongly expressed at higher engagement. The mechanism remains inferential, but sustained practice, feedback use, and strategy revision provide plausible processes through which students with weaker prior standing can reduce knowledge gaps.

The interaction explained an additional 2.1% of achievement variance and had a standardized coefficient of −0.144. This increment is modest in individual prediction but potentially relevant for a malleable educational process layered onto admissions information. It does not imply that engagement eliminates prior differences. The high-engagement slope CI [−0.011, 0.210] and the 18.1% of observations above the Johnson–Neyman threshold require the more limited conclusion that the association weakened and became less precisely distinguishable from zero.

#### 5.1.2 How academic self-efficacy is indirectly associated with vocational course achievement through engagement

H2 showed a cross-sectional indirect association from academic self-efficacy through engagement to later-extracted achievement records. Because self-efficacy and engagement were concurrent, the pattern is also compatible with reciprocal influence, prior study habits, or achievement experiences shaping both reports. The objective outcome reduces shared outcome-method bias but does not establish the ordering of the two T1 variables.

This pattern is consistent with the social cognitive sequence of capability beliefs, goal-directed activity, and outcomes (Bandura, 1997; Schunk and DiBenedetto, 2020) and with recent meta-analytic evidence linking self-efficacy to engagement (Liang et al., 2026). A recent article in the same Research Topic found that teacher caring was associated with perceived course-related learning gains through academic self-efficacy and learning engagement (Du et al., 2026). The present study extends that work by controlling standardized admission scores and using objective T2 academic records, thereby moving beyond concurrent self-reported learning gains to an association between psychological resources and subsequent objective achievement. Nevertheless, because self-efficacy and engagement were measured at the same time, we describe an indirect pathway rather than a fully established causal mediation process.

#### 5.1.3 Learning engagement and career adaptability

H3 linked self-efficacy, engagement, and career adaptability within the same T1 survey. The result indicates covariance consistent with the proposed theoretical pathway, not that engagement caused later adaptability. Career planning could increase learning engagement; stable optimism, conscientiousness, or social support could also generate the association. The method-factor sensitivity analysis did not materially attenuate the pattern, but no statistical adjustment can replace temporal separation or independent measurement.

The direct association between academic self-efficacy and career adaptability remained significant after engagement was entered, indicating that engagement explained only part of their association. Beyond learning behavior, efficacy beliefs may relate to adaptability through career-decision confidence, goal persistence, emotion regulation, or cognitive appraisals of uncertainty. Because all three constructs were measured at T1, the observed structure is theoretically consistent but cannot demonstrate that self-efficacy first increased engagement and engagement subsequently increased career adaptability. Even so, the finding suggests that vocational learning and career preparation are not separate processes: stronger participation in current learning may coincide with more developed preparation for future work.

#### 5.1.4 Does teacher support improve the conversion of engagement into achievement?

Teacher support modestly strengthened the engagement–achievement association (standardized interaction = 0.104; Δ*R*^2^ = 0.009). This is consistent with the idea that clear structure, guidance, autonomy support, and relational support may help students direct effort productively. However, sensitivity power was approximately 0.67 and the *p* value was near 0.05. H4 should therefore be treated as preliminary evidence requiring replication, not as a stand-alone basis for a strong intervention claim.

This result is consistent with self-determination theory and the educational-interface framework: when learning environments provide autonomy support, clear structure, and relatedness support, students can understand requirements, receive targeted feedback, and sustain high-quality participation (Ryan and Deci, 2020; Kahu and Nelson, 2018). Whereas recent work treated teacher caring as an antecedent of academic self-efficacy and engagement (Du et al., 2026), the present findings suggest that teacher support may also strengthen the translation of engagement into objective achievement. The interaction contributed an incremental 0.9% of explained variance. The effect was stable but modest, so teacher support should not be interpreted as a single determining condition of academic performance.

### 5.2 Theoretical contributions

First, the study extends engagement research by testing the established protective-model logic with multisource admissions and achievement records in vocational higher education. The contribution is contextual and empirical: it does not propose “protective moderation” as a new statistical construct.

Second, the study identifies a psychological–behavioral–outcome pathway from capability beliefs through learning engagement to subsequent objective achievement. Compared with research based on concurrent self-reported course gains (Du et al., 2026), the present study controlled standardized admission scores and used T2 achievement from academic records, reducing the influence of shared time and method on the principal academic outcome.

Third, the integrated model clarifies that engagement can occupy distinct statistical roles across equations. The admission score × engagement interaction remained after self-efficacy, teacher support, and the engagement × support interaction were entered together, while the conditional indirect association also remained. These roles are compatible but remain associational.

Fourth, teacher support and career adaptability extend the contextual and developmental meaning of the model. The moderation by teacher support shows that individual engagement is more readily converted into objective achievement in a supportive instructional environment. The career adaptability result suggests that engagement in current vocational learning is not fully separate from preparation for future careers. By interpreting concurrent T1 variables only as indirect associations, the study also distinguishes theoretically plausible mechanisms from inferences supported by the present design.

### 5.3 Practical implications

Vocational colleges should avoid treating admission scores as immutable labels. The present interaction was small, so it supports adding engagement information to a broader support assessment rather than replacing admissions indicators or mandating a particular intervention. Students with low prior standing and low engagement may merit closer assessment, but decisions should combine multiple academic and contextual indicators.

Self-efficacy-oriented practices—mastery experiences, clear feedback, strategy instruction, and manageable progression—are plausible targets for future trials. The present cross-sectional indirect association does not show that increasing self-efficacy will necessarily raise engagement or grades; intervention studies should test that causal sequence directly.

Teacher support should be viewed as a candidate contextual facilitator. Because H4 explained less than 1% additional variance and had limited sensitivity, the result justifies replication and experimental evaluation of specific practices, not a claim that support alone determines whether engagement becomes achievement.

Career adaptability can be integrated into vocational curricula and practical teaching rather than being confined to separate career-guidance activities. Industry problems, authentic projects, internship reflection, occupational exploration, and career-situated tasks can help students connect current learning with future occupational roles, allowing engagement to support both vocational competence and career preparation. Earlier career exploration and capability feedback may be particularly useful for students with lower academic self-efficacy, creating an integrated system of academic and career support.

### 5.4 Limitations and future directions

Several limitations qualify the findings. First, the sample came from one Chinese vocational college, 11 majors, and three institution-specific admission pathways (Wang and Liu, 2026). The results generalize most directly to comparable Chinese vocational institutions using similar admissions and grading systems, not to universities, other countries, or vocational systems with different selection rules. A multisite replication should prespecify the H1 interaction, recruit institutions across regions and institutional tiers, harmonize course-level records, and test institution and pathway heterogeneity.

Second, all four T1 measures were contextually adapted and used agreement ratings. No expert content-validity review, cognitive interviewing, pilot sample, or independent cross-validation was conducted. HTMT and Fornell–Larcker results supported discriminant validity, but AVE was low and the first-year subgroup showed weaker configural fit. Formal metric and scalar invariance were not established. The exact administered bilingual items are provided in Supplementary Table S1b to support transparency, but independent psychometric replication remains essential.

Third, H2 and H3 do not establish causal mediation. The T1 constructs were concurrent, the 14–45-day interval refers to record extraction rather than a fully nonoverlapping achievement period, and H3 is entirely self-reported. Reverse ordering, reciprocal influence, common response processes, and unmeasured confounding remain plausible. Although the latent method-factor sensitivity model did not materially alter the H3 estimate, the absence of a marker variable and the shared measurement occasion mean that common method variance cannot be excluded. Fourth, course fixed effects and within-course standardization reduced course-level concerns, but instructor identifiers were unavailable; instructor-specific grading cannot be separated. Fifth, the study was not preregistered. Although H1–H4 were specified before formal testing, independent preregistered replication is needed, especially for the small H4 interaction.

## 6 Conclusion

In 491 vocational college students, learning engagement negatively moderated the admission-score–achievement association in a pattern consistent with the protective model. The interaction was stable after integrating the focal equations, controlling course count, standardizing within course code, estimating course fixed effects, and examining admission pathways. It weakened rather than erased the admission-score association. The self-efficacy indirect pathways were cross-sectional, and the teacher-support interaction was small.

Admission scores therefore describe relative prior standing but not a fixed developmental ceiling. The present evidence supports engagement as a modest boundary condition in this sample, while causal, measurement, and generalization claims await preregistered multisite and longitudinal replication.

## Data Availability

The datasets presented in this article are not readily available because the dataset contains student-level information matched across questionnaire responses, institutional admissions records, and academic records. Public release is restricted by participant privacy protections, institutional data-governance requirements, and the conditions of the ethics approval. De-identified data and analytical materials may be accessed only following a reasonable request and approval by the authors' institution and the relevant ethics review body. Requests to access the datasets should be directed to Maoguo Ye, yemaoguo@hypt.edu.cn.
