# Supplementary material for: Learning engagement as a protective moderator of the admission score–vocational course achievement association
Source: Front Psychol. 2026 Oct 7;17:1971032. doi: 10.3389/fpsyg.2026.1971032 (PMC13684422; doi:10.3389/fpsyg.2026.1971032)
Supplement: Supplementary file 1 [file Supplementary_File_1.docx]

**Supplementary Material**

# Supplementary Table S1a. Administered measures and adaptation transparency

| **Construct** | **Codes** | **Dimensions** | **Administered response** | **Status** |
| --- | --- | --- | --- | --- |
| Academic self-efficacy | ASE1–ASE8 | Course efficacy (1–4); self-regulatory efficacy (5–8) | 5-point agreement | Context-adapted; no separate pilot/expert review |
| Learning engagement | LE1–LE9 | Vigor (1–3); dedication (4–6); absorption (7–9) | 5-point agreement | UWES-informed wording; frequency format changed |
| Career adaptability | CA1–CA12 | Concern, control, curiosity, confidence (3 each) | 5-point agreement | CAAS-SF-informed; capability format changed |
| Teacher support | TS1–TS9 | Autonomy, structure, relatedness (3 each) | 5-point agreement | TASC-informed context-specific item set; no separate content-validity study |

# Supplementary Table S1b. Exact administered bilingual items

| **Code** | **Construct** | **Dimension** | **Exact Chinese wording administered** | **Checked English translation** |
| --- | --- | --- | --- | --- |
| ASE1 | Academic self-efficacy | Course efficacy | 我有信心理解本专业课程中的核心概念和重点内容。 | I am confident that I can understand the core concepts and key content in the courses for my major. |
| ASE2 | Academic self-efficacy | Course efficacy | 即使专业课程比较难，我也相信自己能够学会。 | Even when the courses in my major are difficult, I believe that I can learn the material. |
| ASE3 | Academic self-efficacy | Course efficacy | 我有能力完成本专业课程中的作业、报告或项目任务。 | I am capable of completing assignments, reports, or project tasks in the courses for my major. |
| ASE4 | Academic self-efficacy | Course efficacy | 遇到专业学习中的困难时，我能够找到办法解决。 | When I encounter difficulties in my major studies, I can find ways to solve them. |
| ASE5 | Academic self-efficacy | Self-regulatory efficacy | 我能够按照学习计划完成专业课程学习任务。 | I can complete the learning tasks for my major courses according to my study plan. |
| ASE6 | Academic self-efficacy | Self-regulatory efficacy | 我能够坚持课前预习、课后复习或整理学习笔记。 | I can consistently preview lessons before class, review after class, or organize my study notes. |
| ASE7 | Academic self-efficacy | Self-regulatory efficacy | 即使学习任务较多，我也能合理安排时间完成。 | Even when I have many learning tasks, I can manage my time effectively to complete them. |
| ASE8 | Academic self-efficacy | Self-regulatory efficacy | 我能够主动利用教材、网络资源或教师指导来改进学习。 | I can actively use textbooks, online resources, or teacher guidance to improve my learning. |
| LE1 | Learning engagement | Vigor | 在专业学习中，我通常感到精力充沛。 | In my major studies, I usually feel energetic. |
| LE2 | Learning engagement | Vigor | 面对专业学习任务时，我愿意投入较多努力。 | When facing learning tasks in my major, I am willing to invest substantial effort. |
| LE3 | Learning engagement | Vigor | 即使学习中遇到困难，我也能坚持下去。 | Even when I encounter difficulties in learning, I can persist. |
| LE4 | Learning engagement | Dedication | 我认为专业学习对自己的未来发展很有意义。 | I believe that studying my major is meaningful for my future development. |
| LE5 | Learning engagement | Dedication | 我对本专业的学习内容感到有兴趣。 | I am interested in the learning content of my major. |
| LE6 | Learning engagement | Dedication | 我为自己能够学习本专业知识和技能感到自豪。 | I am proud to be learning the knowledge and skills of my major. |
| LE7 | Learning engagement | Absorption | 学习专业课程时，我能够较长时间保持注意力。 | When studying courses in my major, I can maintain my attention for a relatively long time. |
| LE8 | Learning engagement | Absorption | 当我投入专业学习时，常常会忘记时间。 | When I am engaged in studying my major, I often lose track of time. |
| LE9 | Learning engagement | Absorption | 我能够沉浸在专业课程、实训或项目学习中。 | I can become immersed in major courses, practical training, or project-based learning. |
| CA1 | Career adaptability | Concern | 我会主动思考自己未来的职业发展方向。 | I actively think about the direction of my future career development. |
| CA2 | Career adaptability | Concern | 我会关注未来就业市场和行业发展变化。 | I pay attention to changes in the future job market and industry development. |
| CA3 | Career adaptability | Concern | 我会提前为毕业后的就业或升学做准备。 | I prepare in advance for employment or further study after graduation. |
| CA4 | Career adaptability | Control | 我认为自己的职业发展需要由自己主动负责。 | I believe that I should take active responsibility for my own career development. |
| CA5 | Career adaptability | Control | 面对职业选择时，我会主动做出决定。 | When faced with career choices, I take the initiative to make decisions. |
| CA6 | Career adaptability | Control | 我愿意为实现自己的职业目标付出努力。 | I am willing to make efforts to achieve my career goals. |
| CA7 | Career adaptability | Curiosity | 我愿意了解不同岗位、行业或职业发展路径。 | I am willing to learn about different positions, industries, or career development paths. |
| CA8 | Career adaptability | Curiosity | 我会主动探索本专业相关的职业机会。 | I actively explore career opportunities related to my major. |
| CA9 | Career adaptability | Curiosity | 我愿意尝试新的学习、实习或职业体验机会。 | I am willing to try new learning, internship, or career-experience opportunities. |
| CA10 | Career adaptability | Confidence | 我相信自己能够应对未来求职中的困难。 | I believe that I can cope with difficulties in future job seeking. |
| CA11 | Career adaptability | Confidence | 我相信自己能够胜任未来职业中的基本任务。 | I believe that I can competently perform the basic tasks of my future occupation. |
| CA12 | Career adaptability | Confidence | 即使未来职业环境发生变化，我也有信心适应。 | Even if the future work environment changes, I am confident that I can adapt. |
| TS1 | Teacher support | Autonomy | 教师尊重我在专业学习中的想法和选择。 | Teachers respect my ideas and choices in my major studies. |
| TS2 | Teacher support | Autonomy | 教师鼓励我主动思考和解决学习问题。 | Teachers encourage me to think independently and solve learning problems. |
| TS3 | Teacher support | Autonomy | 教师会给我一定空间，让我自主完成学习任务。 | Teachers give me some latitude to complete learning tasks independently. |
| TS4 | Teacher support | Structure | 教师能够清楚说明课程目标和学习要求。 | Teachers clearly explain course objectives and learning requirements. |
| TS5 | Teacher support | Structure | 当我遇到学习困难时，教师会提供有效指导。 | When I encounter learning difficulties, teachers provide effective guidance. |
| TS6 | Teacher support | Structure | 教师会帮助我理解专业知识与实际工作的联系。 | Teachers help me understand the connection between knowledge in my major and actual work. |
| TS7 | Teacher support | Relatedness | 教师关心我在学习中的困难和进步。 | Teachers care about my difficulties and progress in learning. |
| TS8 | Teacher support | Relatedness | 教师的鼓励让我更有信心学好专业课程。 | Teachers' encouragement gives me greater confidence in learning my major courses well. |
| TS9 | Teacher support | Relatedness | 我能感受到教师对学生学习发展的重视。 | I can sense that teachers value students' learning and development. |

Note. The Chinese wording is reproduced from the questionnaire administered to participants. The English translations were checked for semantic fidelity for reporting purposes. All items used a 5-point agreement scale from 1 (strongly disagree) to 5 (strongly agree). No separate expert review, cognitive interview, pilot sample, or measurement-equivalence study was conducted.

# Supplementary Table S2. Fornell–Larcker matrix and HTMT ratios

| **Construct** | **ASE** | **LE** | **CA** | **TS** |
| --- | --- | --- | --- | --- |
| ASE | 0.600 |  |  |  |
| LE | 0.494 | 0.577 |  |  |
| CA | 0.335 | 0.410 | 0.576 |  |
| TS | 0.103 | 0.244 | 0.279 | 0.572 |

Note. Diagonal values are square roots of item-level overall-construct AVE; off-diagonal values are observed composite correlations. Each diagonal exceeded the corresponding correlations.

| **HTMT** | **ASE** | **LE** | **CA** | **TS** |
| --- | --- | --- | --- | --- |
| ASE | 1.000 |  |  |  |
| LE | 0.604 | 1.000 |  |  |
| CA | 0.401 | 0.489 | 1.000 |  |
| TS | 0.134 | 0.301 | 0.336 | 1.000 |

All off-diagonal HTMT values were below the conservative .85 threshold.

# Supplementary Table S3. Item-level and common-method-factor sensitivity analyses

| **Model** | **χ²** | **df** | **CFI** | **TLI** | **RMSEA** | **Interpretation** |
| --- | --- | --- | --- | --- | --- | --- |
| 12 correlated item-level factors | 595.855 | 599 | 1.000 | 1.000 | .000 | No correlated residuals or item deletion |
| 12 factors + orthogonal equal-loading method factor | 592.452 | 598 | 1.000 | 1.000 | .000 | ΔCFI < .001; ΔRMSEA = .000; AIC/BIC increased |
| Single self-report factor | 3,309.638 | 665 | .502 | .473 | .090 | Poor fit |

H3 factor-score path-product estimate: .271 without and .284 with the method factor. This does not rule out common method bias; it shows that this particular adjustment did not attenuate the pattern.

| **Subgroup** | **n** | **CFI** | **TLI** | **RMSEA** |
| --- | --- | --- | --- | --- |
| Male | 221 | .989 | .988 | .014 |
| Female | 270 | .989 | .987 | .014 |
| First year | 78 | .894 | .875 | .048 |
| Second year | 194 | .975 | .971 | .022 |
| Third year | 219 | .961 | .954 | .027 |

These are configural subgroup fits only. No equality constraints were tested; metric/scalar invariance is not claimed. The first-year group was small relative to the 38-item, 12-factor model and showed weaker fit.

# Supplementary Table S4. Integrated model and conditional indirect associations

| **Focal term** | **B** | **HC3 SE** | **p** | **95% CI** |
| --- | --- | --- | --- | --- |
| Admission score × engagement | −.265 | .067 | < .001 | [−.396, −.133] |
| Engagement × teacher support | .318 | .146 | .030 | [.031, .605] |
| Academic self-efficacy direct effect | .103 | .087 | .235 | [−.067, .273] |

| **Condition** | **Effect/index** | **BootSE** | **95% bootstrap CI** |
| --- | --- | --- | --- |
| Teacher support −1 SD | 0.225 | 0.054 | [0.123, 0.335] |
| Teacher support mean | 0.310 | 0.047 | [0.219, 0.402] |
| Teacher support +1 SD | 0.394 | 0.066 | [0.267, 0.525] |
| Index of moderated mediation | 0.156 | 0.070 | [0.012, 0.291] |

# Supplementary Table S5. Course-level and pathway sensitivity analyses for H1

| **Specification** | **Analytic units** | **Interaction B** | **SE** | **p** | **95% CI** |
| --- | --- | --- | --- | --- | --- |
| Course fixed effects; student-clustered SE | 2,488 course records; 66 codes | −1.546 | .377 | < .001 | [−2.287, −.805] |
| Course-standardized credit-weighted student outcome | 491 students | −.267 | .069 | < .001 | [−.403, −.131] |
| Course-count control | 491 students | −.263 | .069 | < .001 | [−.397, −.128] |

| **Pathway** | **n** | **Interaction B** | **HC3 SE** | **p** | **95% CI** |
| --- | --- | --- | --- | --- | --- |
| National College Entrance Examination | 176 | -0.293 | 0.129 | 0.025 | [-0.548, -0.038] |
| Spring Academic Proficiency Test | 174 | -0.128 | 0.127 | 0.315 | [-0.379, 0.123] |
| 3+Certificate | 141 | -0.383 | 0.114 | 0.001 | [-0.608, -0.157] |

Joint robust Wald test of the two pathway differences: F(2, 476) = 1.265, p = .283.

# Supplementary Table S6. Interaction sensitivity and Johnson–Neyman coverage

| **Interaction** | **ΔR²** | **f²** | **Estimated power** | **Standardized effect (95% boot CI)** | **Coverage** |
| --- | --- | --- | --- | --- | --- |
| H1: admission score × engagement | .021 | .028 | .957 | −.144 [−.214, −.071] | JN 4.09; 89 students (18.1%) above |
| H4: engagement × support | .009 | .012 | .666 | .104 [.014, .195] | JN 2.53; 479 students (97.6%) at/above |

At N = 491 and α = .05, the minimum detectable incremental f² was .016 for 80% power and .021 for 90% power. H4 therefore had limited sensitivity and should be treated as replication-sensitive.

# Supplementary Table S7. Variable construction and standardization specification

| **Variable** | **Construction** | **Grouping/interpretation** |
| --- | --- | --- |
| ADM_Z | (ADM_RAW − group mean)/group SD | Admission year × admission pathway × examination category/scoring system |
| PROF_RAW | Σ(grade × credit)/Σcredit | Valid vocational courses; 4–6 per student |
| PROF_Z | (PROF_RAW − group mean)/group SD | Admission year × academic major |
| COURSE_Z | (course grade − course-code mean)/course-code SD | Exact COURSE_CODE; revision sensitivity outcome |
| COURSE_Z_AGG | Σ(COURSE_Z × credit)/Σcredit | Student-level course-adjusted sensitivity outcome |
| H1 interaction | mean-centered ADM_Z × mean-centered LE_MEAN | Main effects conditional at moderator/predictor means |
| H4 interaction | mean-centered LE_MEAN × mean-centered TS_MEAN | Main effects conditional at moderator/predictor means |

# Analytical materials

The revision package includes an executed notebook, Python scripts, and corrected SPSS/PROCESS 4.2 syntax reproducing linkage checks, HTMT/Fornell–Larcker matrices, item-level CFA, method-factor sensitivity, integrated regression, course fixed effects, within-course standardization, pathway analyses, bootstrap confidence intervals, sensitivity power, alternative-outcome robustness checks, and revised figures. Primary and integrated PROCESS models use fixed seed 20260830; extended-covariate models use fixed seed 20260831; alternative-outcome models use fixed seed 20260832. Student identifiers and row-level data are not included in the public-facing package.
